# Predictive factors and adalimumab efficacy in managing chronic recurrence Vogt-Koyanagi-Harada disease

**DOI:** 10.1186/s12886-024-03511-9

**Published:** 2024-06-07

**Authors:** Hui Feng, Weixin Chen, Jianzhu Yang, Haorong Kong, Hongyu Li, Yuan He, Hong Wang

**Affiliations:** Beijing Ophthalmology and Visual Sciences Key Laboratory, Beijing Tongren Hospital, Beijing Tongren Eye Center, Beijing Institute of Ophthalmology, Capital Medical University, Beijing, China

**Keywords:** Predictive factor, Prognosis, Uveitis, Vogt–Koyanagi–Harada disease, Adalimumab

## Abstract

**Background:**

This study explores prognostic factors influencing Vogt-Koyanagi-Harada (VKH) disease and observes the efficacy and safety of Adalimumab (ADA) in treating recurrence in Vogt-Koyanagi-Harada (VKH) patients.

**Methods:**

A retrospective study was conducted on all patients diagnosed with VKH disease at Beijing Tongren Hospital between 2020 and 2023. Clinical data included initial and final visual acuity, age, gender, ocular complications, treatment modalities, disease duration, and recurrence frequency.

**Results:**

A total of 62 VKH patients were included, comprising 34 in the acute-resolved group and 28 in the chronic-recurrent group. The mean age of patients in the acute-resolved group was 38.29 ± 15.46 years, while the mean age of chronic-recurrent group had a 49.00 ± 16.43 years. Initial best-corrected visual acuity (BCVA) examination at the first visit showed an average BCVA of 0.64 ± 0.29 logMAR in the acute-resolved group and 1.38 ± 0.54 logMAR in the chronic-recurrent group (*p* = 0.002). During follow-up, ocular complications were observed in 29.4% of the acute-resolved group patients and 41.7% of the chronic-recurrent group patients (*P* = 0.006). “Sunset glow fundus” was observed in 23.5% of the acute-resolved group and 64.3% of the chronic-recurrent group patients (*P* = 0.001). Poor initial BCVA (*P* = 0.046) and the occurrence of “sunset glow fundus” (*P* = 0.040) were significantly associated with progression to the chronic recurrent phase. Logistic regression analysis revealed that older age at onset (*P* = 0.042) and the occurrence of “sunset glow fundus” (*P* = 0.037) were significant predictors for progression to the chronic recurrent phase. ADA significantly reduced anterior chamber inflammatory cells (*P* = 0.000) and vitreous cavity inflammatory cells (*P* = 0.001) in the chronic-recurrent group, and markedly decreased the recurrence rate in VKH patients (*P* = 0.009).

**Conclusion:**

In comparison to acute-resolved patients, chronic-recurrent patients exhibited poorer initial BCVA and a significantly increased incidence of “sunset glow fundus.” Older age at onset and the occurrence of “sunset glow fundus” at diagnosis are crucial predictive factors for VKH patients progressing to the chronic recurrent phase. ADA effectively alleviates refractory VKH disease and is generally well-tolerated.

## Introduction

Vogt-Koyanagi-Harada (VKH) is an autoimmune disease characterized by severe bilateral non-granulomatous panuveitis, typically accompanied by systemic symptoms such as tinnitus, alopecia, myelitis, and vitiligo [1]. Patients with VKH may experience serious visual impairment due to ocular inflammation and its complications, including cataracts, secondary glaucoma, and choroidal neovascularization [2–4].

VKH disease is clinically divided into four stages: prodromal, uveitic, convalescent, and chronic recurrent [5, 6]. The treatment approach for the acute phase of VKH involves prolonged and aggressive use of systemic corticosteroids, often combined with systemic immunomodulatory therapy. Some VKH patients, when promptly and appropriately treated with systemic corticosteroids and immunomodulatory therapy, may gradually reduce the dosage of systemic corticosteroids without worsening the condition [7, 8]. However, there is a subset of VKH patients who, even with proper and aggressive use of systemic corticosteroids and immunomodulatory therapy, may experience disease recurrent. In cases of VKH disease recurrence, inflammation becomes difficult to control, necessitating additional therapeutic approaches such as increasing corticosteroid doses, combining various immunosuppressive agents, or introducing biologic agents [9–11]. While a small number of studies have reported various factors associated with the progression of VKH to the chronic recurrent stage, such as age at onset, poor initial visual acuity, rapid corticosteroid tapering, and disease presentation type (e.g., optic disc edema or serous retinal detachment) [10, 12, 13], larger studies exploring the influencing factors on VKH progression to the chronic recurrent stage are still lacking. Therefore, recognizing potential prognostic factors influencing disease recurrence and adjusting treatments accordingly holds significant clinical importance.

Tumor necrosis factor-alpha (TNF-α) has played a crucial role in the development of chemokines, adhesion molecules, and cytokines associated with ocular inflammation [14, 15]. In the management of severe posterior uveitis and panuveitis, the application of TNF-α inhibitors stands prominent. The guidelines offered by the American Uveitis Society suggested TNF-α inhibitors as prospective second-line immunosuppressive agents to control over the intense ocular inflammation [16]. The efficacy of Adalimumab (ADA; AbbVie, North Chicago, IL, USA) treatment for VKH patients resistant to conventional therapy has been demonstrated. Yang et al. evaluated nine refractory VKH patients treated with ADA in combination with corticosteroids and immunosuppressive agents for an average duration of 10 months [11]. During the treatment, most patients exhibited good control of ocular inflammation, including anterior chamber and vitreous inflammation. Due to the small sample size, long-term and larger-scale observations are still needed to assess the safety of ADA.

Therefore, we conducted this retrospective study to identify potential risk factors influencing the prognosis of VKH patients. These factors can assist in optimizing treatment and predicting whether VKH patients initially treated with corticosteroids and immunomodulatory therapy will require further intervention in the future. To achieve this, the study reviewed ocular examination results, complication occurrences, and follow-up information for all VKH patients seen at our institution. We compared ocular data between VKH patients who responded to conventional treatment during the acute phase of initial onset and those who progressed to the chronic recurrent stage despite conventional treatment being ineffective. Additionally, we assessed the efficacy and safety of ADA treatment for this subset of refractory VKH patients.

## Methods

### Patients and diagnosis

Clinical records of all VKH patients attending the clinic at Beijing Tongren Hospital, Capital Medical University from January 2020 to December 2023 were reviewed by researchers. VKH disease patients included in the study devoid of any history of ocular trauma or intraocular surgery, and undergoing treatment at our clinic. The study was approved by the Institutional Research and Ethics Committee of Beijing Tongren Hospital and followed the principles of the Helsinki Declaration. Written informed consent was obtained from adult patients and their parents or legal guardians before enrollment.

### Clinical examination

All VKH patients underwent comprehensive ophthalmic examinations, including best-corrected visual acuity (BCVA), anterior segment examination with a slit-lamp, intraocular pressure (IOP), and indirect ophthalmoscopy. In addition, relevant imaging studies were performed, including fundus photography, B-scan ultrasound, fluorescein angiography (FFA), indocyanine green angiography (ICGA), and SD-OCT. We also recorded treatment details, systemic diseases, ocular complications, and follow-up times for all patients. Anterior segment inflammation was assessed according to the Standardization of Uveitis Nomenclature (SUN) criteria [17], and the vitreous inflammation was assessed according to criteria developed by RB Nussenblatt [18]. 

BCVA was assessed using the standard logarithmic visual acuity chart and converted to the LogMAR format. For patients with visual function abnormalities such as “counting fingers/hand motion/light perception,” BCVA was quantified to facilitate statistical analysis [19].

We define inactive VKH uveitis as eyes without active inflammation in the choroid or retina, with anterior chamber cell grading of 0.5 + or lower, or vitreous haze of 0.5 + or lower. Recurrence of inflammation is defined as an increase of two grades in anterior chamber cell grading, the appearance of mutton-fat keratic precipitates in the posterior cornea, or an increase of two grades in vitreous haze grading, or the appearance of new active inflammatory choroidal or retinal vascular lesions (as determined by clinical examination or auxiliary examinations such as FFA) [17, 18]. 

Additionally, the laboratory workup was conducted for all patients to exclude infection and evaluate systemic conditions, which included routine blood and urine tests, liver and kidney function analysis, infectious disease investigations (including hepatitis serology, and screening for syphilis and HIV antibodies). Each patient underwent tuberculosis screening through both the Purified Protein Derivative (PPD) and QuantiFERON-TB Gold test (QFT).

### Treatment

During the acute phase, the oral corticosteroid tapering protocol initiated with an initial dosage of 1 mg/kg per day, subsequently decreasing by 5 mg every 7 days until reaching a daily dosage of 20 mg. Subsequently, a reduction by 2.5 mg every 14 days ensued until reaching a daily dosage of 15 mg. Thereafter, a progressive tapering of 2.5 mg occurred at intervals of 1.5 months until reaching a final daily dosage of 10 mg. Finally, a bi-monthly reduction of 2.5 mg was implemented [20]. In addition to oral prednisolone, immunosuppressive therapy was administered concurrently. For example, cyclosporine at a daily dose of 2.5 mg/kg or mycophenolate mofetil 1.5 g/d.

If conventional immunosuppressive therapy fails to control ocular inflammation even with the combination of oral prednisolone and immunosuppressive agents, adalimumab can be added. Adalimumab is initially received subcutaneous adalimumab 80 mg. Two weeks post the initial administration, a subcutaneous injection of 40 mg was administered every two weeks. ADA was exclusively administered to VKH patients with negative interferon-gamma release assay tests and/or tuberculin skin tests.

Patients were divided into two subgroups: those who achieved control using only steroids and immunosuppressive agents during the acute phase, with no inflammation recurrence during follow-up (Group 1: Acute-resolved group), and those who controlled uveitis with steroids and immunosuppressive agents during the acute phase but experienced recurrent inflammation during follow-up, requiring the addition of ADA for inflammation control (Group 2: Chronic-recurrent group).

### Statistical analysis

Statistical analyses were performed using the commercially available SPSS version 22.0 (IBM, Chicago, USA). First, the continuous variables were described as means and standard errors of the mean (SEM). The two groups were compared using the independent-sample t-test and Mann–Whitney statistical test. The categorical variables were described as frequencies and constituent ratios and were analysed using the chi-square test. Multivariate Logistic model was performed stepwise. Differences were considered statistically significant at *P*-values of ≤ 0.05.

## Results

A total of 124 eyes from 62 VKH patients were examined. Among them, 43 patients were female (69.4%), and 19 patients were male (30.6%). All patients were in the acute phase during their initial visit and received initial treatment at our clinic. 34patients (54.8%) showed no sustained or recurrent inflammation during follow-up (Acute-resolved group), while 28 patients (45.2%) exhibited sustained or recurrent inflammation during follow-up (Chronic-recurrent group). The mean age of the acute-resolved group was 38.29 ± 15.46 years, while the chronic-recurrent group had a mean age of 49.00 ± 16.43 years. Initial BCVA examination at the first visit revealed an average BCVA of 0.64 ± 0.29 logMAR in the acute-resolved group and 1.38 ± 0.54 logMAR in the chronic-recurrent group (*p* = 0.002). After systemic treatment, the BCVA in both groups improved to 0.18 ± 0.26 logMAR and 0.39 ± 0.27 logMAR, respectively (*p* = 0.627). The average follow-up period was 27.72 ± 11.29 months for the acute-resolved group and 34.29 ± 10.37 months for the chronic-recurrent group. Although there was no significant difference in follow-up time between the two groups, it was observed that patients progressing to the chronic phase had a longer follow-up time. The demographic and clinical data for each group are presented in Table 1.

**Table 1:** Demographics and baseline clinical of patients in acute-resolved group and chronic-recurrent group

Characteristic	Acute-resolved group (*N* = 34)	Chronic -recurrent group (*N* = 28)	*P* value	
Age (years)	38.29 ± 15.46	49.00 ± 16.43	0.178	
Sex (Male/Female)	10/24	9/19	0.739	
BCVA at present (LogMAR)	0.64 ± 0.29	1.38 ± 0.54	0.002	
BCVA at final visit (LogMAR)	0.18 ± 0.26	0.39 ± 0.27	0.627	
Sunset glow fundus during follow-up, no (%)	8 (23.5%)	18 (64.3%)	0.001	
Follow-up time (months)	27.72 ± 11.29	34.29 ± 10.37	0.539	
Ocular finding at baseline				
Anterior chamber cells, no (%)	4 (11.8%)	8 (28.6%)	0.096	
Vitreous cells, no (%)	9 (26.5%)	11 (39.3%)	0.283	
Posterior synechiae, no (%)	8 (23.5%)	10 (35.7%)	0.293	
Exudative retinal detachment, no (%)	26 (76.5%)	24 (85.7%)	0.395	
At least one complication during follow-up, no (%)	10 (29.4%)	18 (41.7%)	0.006	
Cataract, no (%)	6 (17.6%)	10 (39.3%)	0.106	
Glaucoma, no (%)	3 (8.8%)	4 (14.3%)	0.526	
Choroidal neovascularization, no (%)	1 (2.9%)	4 (14.3%)	0.103	

The initial eye examination findings at the first visit are presented in Table 1. The most common ocular signs in both groups were vitreous inflammatory cells and exudative retinal detachment. Exudative retinal detachment was detected in 26 patients (76.5%) in the acute-resolved group and in 24 patients (85.7%) in the chronic-recurrent group. Vitreous inflammatory cells were found in 9 patients (26.5%) in the acute-resolved group and in 11 patients (39.3%) in the chronic-recurrent group. No statistically significant differences were observed between the two groups in any ocular examination results.

We also documented the occurrence of ocular complications during the follow-up period. As shown in Table 1, at least one complication was observed in 10 patients (29.4%) in the acute-resolved group and 18 patients (41.7%) in the chronic-recurrent group. The difference between the two groups was statistically significant, with a higher frequency of ocular complications in the chronic-recurrent group. The most common ocular complication was cataract, occurring in 17.6% and 39.3% of patients in the acute-resolved and chronic-recurrent groups, respectively. Additionally, there was a significant difference in the frequency of “sunset glow fundus” during the follow-up between the two groups. “Sunset glow fundus” was observed in 8 patients (23.5%) in the acute-resolved group and 18 patients (64.3%) in the chronic-recurrent group.

Table 2 displays details of systemic treatment for VKH disease patients at baseline and the last follow-up. Initial treatment for all patients included steroids and immunosuppressive agents. The baseline oral corticosteroid dose in the acute-resolved group and chronic-recurrent group was 54.44 ± 7.05 mg and 53.33 ± 7.79 mg, respectively. At the last follow-up, the oral corticosteroid dose in both groups significantly decreased to 3.18 ± 2.69 mg and 2.43 ± 2.78 mg. The most commonly preferred immunosuppressive treatment in both groups was Cyclosporine A. At the last follow-up, there was no significant difference in the oral corticosteroid and immunosuppressive agent doses between the two groups. No severe adverse events related to Cyclosporine A or Mycophenolate mofetil treatment occurred during the follow-up in this study.

**Table 2:** Category and dosage of systemic treatments between the groups at the baseline and final visit

Group	Acute-resolved group (*N* = 34)	Chronic -recurrent group (*N* = 28)	*P* value	
Systemic treatment at baseline, patients, no (%)				
Oral glucocorticoids	34	28	-	
Cyclosporine A	26(76.5%)	24(85.7%)	0.359	
Mycophenolate mofetil	8(23.5%)	4(14.3%)	0.359	
Dosage at baseline, mean ± SD				
Oral glucocorticoids, mg qd	54.44 ± 7.05	53.33 ± 7.79	0.685	
Cyclosporine A, mg bid	56.25 ± 6.68	60.00 ± 5.37	0.641	
Mycophenolate mofetil, g bid	0.65 ± 0.10	0.69 ± 0.09	0.460	
Dosage at the final visit, mean ± SD				
Oral glucocorticoids, mg qd	3.18 ± 2.69	2.43 ± 2.78	0.590	
Cyclosporine A, mg bid	15.00 ± 5.27	27.50 ± 7.91	0.107	
Mycophenolate mofetil, g bid	0.25	0.25	1.000	

To further analyze factors associated with progression to the chronic-recurrent phase, we conducted both univariate and multivariate logistic regression analyses. In the univariate analysis, clinical features significantly correlated with recurrent VKH were lower baseline BCVA (*P* = 0.046) and the occurrence of “sunset glow fundus” during follow-up (*P* = 0.040) (Table 3). In the multivariate analysis incorporating age, gender, baseline BCVA, BCVA at the last follow-up, and the occurrence of “sunset glow fundus” during follow-up, older age (OR: 1.063, *P* = 0.042) and the occurrence of “sunset glow fundus” (OR: 1.451, *P* = 0.037) were identified as significant risk factors for recurrent VKH (Table 3).

**Table 3:** Univariate and multivariate logistic regression of prognostic factors for VKH with chronic recurrence features

	Univariate analysis			Multi-variate analysis		
Characteristic	Odds ratio (95% CI)	*P* value	Odds ratio (95% CI)		*P* value	
Age	1.024 (0.985 to 1.064)	0.227	1.063 (1.002 to 1.128)		0.042	
Sex	0.328 (0.317 to 4.211)	0.628	0.874 (0.138 to 5.552)		0.886	
BCVA at present	0.354 (0.128 to 0.982)	0.046	0.348 (0.068 to 1.780)		0.205	
BCVA at final visit	0.162 (0.018 to 1.477)	0.106	0.198 (0.010 to 4.016)		0.291	
Sunset glow fundus	1.128 (1.032 to 1.224)	0.040	1.451 (1.009 to 1.892)		0.037	
Follow-up time	0.971 (0.925 to 1.018)	0.221				
Anterior chamber cells	0.420 (0.086 to 2.051)	0.284				
Vitreous cells	0.605 (0.162 to 2.259)	0.455				
Posterior synechiae	0.602 (0.151 to 2.404)	0.472				
Exudative retinal detachment	0.756 (0.178 to 3.199)	0.703				
At least one complication	0.485 (0.132 to 1.785)	0.276				

The changes in ocular parameters before and after the application of ADA are presented in Table 4. Patients in the chronic-recurrent group received ADA injection therapy due to recurrent inflammation after an average of 19.24 ± 7.84 months of corticosteroid and immunosuppressive treatment. The average follow-up period for receiving ADA treatment was approximately 15.05 ± 6.83 months. Following the addition of ADA treatment in the chronic-recurrent group, BCVA improved from 0.43 ± 0.29 LogMAR before treatment to 0.39 ± 0.27 LogMAR at the last visit (*P* = 0.172). The average times of inflammatory relapses per patient decreased from 1.43 ± 1.22 before ADA treatment to 0.36 ± 0.66 after ADA treatment. Before the addition of ADA treatment, the anterior chamber cell grade and vitreous cell grade were at least 2.36 ± 1.13 and 1.33 ± 0.69. After the addition of ADA treatment, the anterior chamber cell grade and vitreous cell grade decreased to 0.84 ± 0.97 (*P* = 0.000) and 0.39 ± 0.50 (*P* = 0.001) at the last follow-up. The macular thickness remained relatively stable before and after the addition of ADA treatment, changing from 216.06 ± 79.3 μm before treatment to 219.28 ± 77.20 μm at the last visit (*P* = 0.590). During the follow-up, three patients discontinued ADA treatment due to the stability of ocular inflammation and the high cost of ADA. No serious adverse reactions requiring clinical intervention or cessation of ADA treatment were reported.

**Table 4:** The changes of the ocular parameters before and after application of ADA in Chronic -recurrent VKH patients. (*N* = 25)

Characteristic	Before application of ADA	After application of ADA	*P* value
Treatment period, (months)	19.24 ± 7.84	15.05 ± 6.83	
BCVA (LogMAR)	0.43 ± 0.29	0.39 ± 0.27	0.172
Anterior chamber cell grade	2.36 ± 1.13	0.84 ± 0.97	0.000
Vitritis grade	1.33 ± 0.69	0.39 ± 0.50	0.001
Central macular thickness, (µm)	216.06 ± 79.3	219.28 ± 77.20	0.590
Relapse times	1.43 ± 1.22	0.36 ± 0.66	0.009

## Discussion

This retrospective study conducted an analysis of multiple factors to identify those associated with the development of VKH patients into the chronic-recurrent phase. The study revealed a significant correlation between poorer baseline BCVA and the occurrence of “sunset glow fundus” with the progression of patients to the chronic-recurrent phase. Logistic regression analysis further identified older age at the time of diagnosis and the occurrence of “sunset glow fundus” as important predictive factors for VKH patients progressing to the chronic-recurrent phase. These findings suggest that VKH patients with older age at onset, worse initial BCVA, and the manifestation of “sunset glow fundus” are at a higher risk of progressing to the chronic-recurrent phase.

The progression of the disease to the chronic recurrent phase is influenced by multiple factors. Consistent with previous research findings, we found that VKH patients who were older at disease onset, had poorer BCVA, and exhibited “sunset glow fundus” during follow-up showed a strong correlation with progression to the chronic recurrent stage. Previous related studies have mentioned the relationship between treatment regimens, age, visual acuity at onset, and ocular manifestations with the progression of VKH to the chronic recurrent phase. Studies by Okunuki et al. [21] and Kaya et al. [12] both found that elderly patients were more likely to develop chronic disease compared to younger patients. Maruyama et al. [22] compared 19 non-recurrent VKH disease cases with 22 recurrent VKH cases and found that lower visual acuity at presentation and more severe anterior chamber inflammation were adverse prognostic factors for VKH patients progressing to the recurrent type. El-Asrar et al. [23] retrospectively evaluated 34 patients with acute resolved VKH and 19 patients with chronic recurrent VKH, revealing a significantly higher incidence of ocular complications such as cataracts, glaucoma, subretinal neovascularization, “sunset glow fundus,” choroidal retinal atrophy, and peripapillary atrophy during follow-up in patients with chronic recurrent VKH compared to those with initial acute VKH. Therefore, we recommend closer follow-up for VKH patients who are older, have poorer BCVA, and exhibit “sunset glow fundus” to prevent progression to the chronic recurrent stage.

In this study, it was found that during the follow-up period, the incidence of ocular complications such as cataracts, glaucoma, and choroidal neovascularization was significantly higher in patients with chronic-recurrent VKH. Among these, cataracts were the most common complication, followed by glaucoma and choroidal neovascularization. Previous studies on complications of VKH reported cataract incidence ranging from 10.5–38%, [10, 24] glaucoma from 6–45%, [2] and choroidal neovascularization from 2.5–15% [25]. Although VKH patients underwent prolonged systemic corticosteroid treatment, which could potentially increase the incidence of cataracts and glaucoma, this study found no significant differences in the dosage of systemic corticosteroids and immunosuppressants between the two groups. This further emphasizes that the increased occurrence of cataracts and glaucoma in the chronic-recurrent group is associated with multiple episodes of inflammatory relapse.

Consistent with prior studies, our observations revealed a notably higher occurrence of the “sunset glow fundus” in patients experiencing chronic-recurrent VKH compared to those with acute-resolved VKH during the follow-up period. However, we also observed that 8 individuals in the acute resolved group developed sunset glow fundus during follow-up. Studies conducted by Keino et al. also indicated a substantial correlation between the presence of chronic ocular inflammation and the manifestation of the “sunset glow fundus” [26]. The appearance of sunset glow fundus in the acute resolved group may be attributed to the presence of “subclinical inflammation” in these patients, despite the absence of significant signs of ocular inflammation. Clinician may have overlooked this subclinical activity due to its concealed nature and the absence of more sensitive examinations such as ICGA, resulting in inadequate treatment to control inflammation, ultimately leading to the development of sunset glow fundus in the long term [27, 28]. Herbort’s team found that 75% of patients (3 out of 4 patients) experienced recurrence of hypofluorescent dark dots on ICGA when treated with low-dose corticosteroids and showed no clinical signs and fluorescein angiographic signs. This suggests that undetected subclinical disease may persist in the absence of ICGA follow-up, which may explain why, despite apparent disease control, the fundus continues to progress towards “sunset glow fundus” [29]. The histopathological examination of eyes exhibiting the “sunset glow fundus” in VKH patients disclosed scattered inflammatory infiltration, predominantly comprising T lymphocytes, within thickened choroids. Notably, there was evident loss of choroidal melanocytes, indicating the persistence of subclinical inflammation during the recovery phase of VKH disease [30]. Moreover, the longer follow-up duration in chronic-recurrent VKH patients in this study logically suggests an increased chance of inflammation relapse with prolonged disease duration, exposing the eyes to the harmful effects of active inflammation for an extended period, thereby increasing the occurrence of complications.

Previous studies have demonstrated the effectiveness of systemic high-dose corticosteroids with or without immunosuppressants in the initial treatment of VKH disease [31]. However, in a significant proportion of cases, this approach fails to prevent the chronic progression of VKH. Similarly, in our study, all enrolled patients received first-line treatment with corticosteroids and immunosuppressants. Although the addition of immunosuppressants allowed patients to reduce the dosage of oral corticosteroids, a subset of patients still progressed to the chronic-recurrent phase. However, in most patients where corticosteroids were gradually tapered, inflammation relapse occurred, particularly when the dosage was reduced to 15–20 mg/day or below. In this chronic progression resulting from delayed or insufficient treatment, controlling inflammation often involves increasing the dosage of corticosteroids and immunosuppressants, but this simultaneously leads to adverse events both systemically and in the eyes.

Our study suggests that adding ADA treatment is highly beneficial in controlling inflammation and reducing the side effects of traditional treatments when corticosteroids and immunosuppressants fail to stabilize VKH. Furthermore, the addition of ADA therapy did not significantly increase the doses of corticosteroids, cyclosporine A, or mycophenolate mofetil in the chronic recurrent group. It significantly lowers the cell grades in the anterior chamber and vitreous, as well as the frequency of inflammation relapse in VKH patients. Likewise, Nakai et al. discovered the efficacy of ADA treatment in VKH patients who exhibited insufficient responses to corticosteroids with or without immunosuppressants, or those unable to sustain conventional immunosuppressive therapy due to side effects. Six months post ADA treatment, VKH patients demonstrated noteworthy reductions in central choroidal thickness, indocyanine green angiography scores, corticosteroid, and cyclosporine doses in comparison to baseline. Additionally, improvements were observed in BCVA and anterior chamber inflammation [32]. Couto et al. documented the clinical effectiveness and safety of ADA treatment in VKH patients facing active inflammation that showed limited response to conventional therapy. Following ADA treatment, 13 out of 14 patients exhibited diminished inflammation, accompanied by a notable reduction in systemic corticosteroid dosage from 20 mg to 4 mg [33]. 

Additionally, Shinagawa et al. [34] found that among VKH cases treated with prednisolone and cyclosporine A, cyclosporine A was discontinued after increasing ADA treatment. The minimum prednisolone dose required to control intraocular inflammation was reduced in all cases after increasing ADA treatment. For VKH patients experiencing inflammation recurrence, increasing corticosteroid doses concurrently increases systemic side effects (such as diabetes, osteoporosis, and gastrointestinal ulcers). Although immunomodulatory agents like cyclosporine A or methotrexate are better tolerated than corticosteroids, they can also lead to side effects including exacerbation or reactivation of infections, hematological disorders, hepatorenal toxicity, and malignancies. Our study suggests that adding ADA therapy in chronic recurrent patients can effectively control inflammation without increasing corticosteroid and immunomodulatory doses.

This study also has some limitations. The retrospective design of the research may introduce constraints in data collection. The retrospective design of the research may introduce constraints in data collection. Many patients had missing data on FFA, ICGA, and EDI-OCT, and some patients missed these examinations during follow-up. Therefore, we could only analyze based on the available data. In our future studies, we will prioritize the collection of FFA, ICGA, and EDI-OCT results in VKH patients to accurately assess the ocular inflammatory status and provide timely appropriate treatment. Moreover, the study’s sample size is relatively small. Long-term studies with a bigger sample size will be needed to confirm the results. Additionally, a part of the subjects included in the study had initially received treatment outside Beijing Tongren Hospital and were subsequently referred to our institution. Upon their clinic visits to our facility, they had already initiated steroid therapy. We promptly introduced immunosuppressive treatment and adhered to a standardized corticosteroid tapering regimen.

In conclusion, this study confirms a significant association between initial BCVA and the occurrence of “sunset glow fundus” with the progression of patients to the chronic-recurrent phase. Larger age at diagnosis and the occurrence of “sunset glow fundus” were identified as important predictive factors for VKH patients progressing to the chronic-recurrent phase. Furthermore, our study indicate that ADA represents a efficacious and secure alternative for VKH patients unresponsive to conventional therapy, efficiently managing inflammation.

## Data Availability

No datasets were generated or analysed during the current study.

## Abbreviations

VKH: Vogt-Koyanagi-Harada
BCVA: best-corrected visual acuity
ADA: Adalimumab
TNF-α: Tumor necrosis factor-alpha
SUN: Standardization of Uveitis Nomenclature
IOP: intraocular pressure
SD-OCT: spectral domain optical coherence tomography
EDI-OCT: enhanced depth imaging optical coherence tomography
FFA: fundus fluorescein angiography
ICGA: indocyanine green angiography
PPD: Purified Protein Derivative
QFT: QuantiFERON-TB Gold test
